# Non‐Secretory Myeloma With Non‐Producing Phenotype Presented as Plasma Cell Leukaemia With Plasmablast Morphology

**DOI:** 10.1002/jha2.70224

**Published:** 2026-01-20

**Authors:** Ke Xu, Ian Proctor

**Affiliations:** ^1^ Department of Haematology University College London Hospitals NHS Foundation Trust, University College London London UK; ^2^ Specialist Integrated Haematology Malignancy Diagnostic Service Health Services Laboratories, University College London Hospitals NHS Foundation Trust London UK; ^3^ Department of Histopathology University College London Hospitals NHS Foundation Trust London UK

**Keywords:** non‐secretary multiple myeloma, plasma cell leukaemia, plasmablast

1

A 58‐year‐old male presented with acute kidney injury and hypercalcaemia. The blood work showed haemoglobin 124 g/L, white blood cells 9.44 × 10^9^/L (neutrophil 2.99 × 10^9^/L, lymphocytes 3.35 × 10^9^/L) and platelets 79 × 10^9^/L. Corrected calcium 3.51 mmol/L (normal range 2.2–2.6 mmol/L), creatinine 308 µmol/L, LDH 383 IU/L (normal range 135–225 IU/L), β2‐microglobulin 12.8 mg/L (normal range 0–2.3 mg/L). There was clear evidence of immunoparesis (IgA 0.21, IgG 3.64 and IgM 0.2 g/L), but paraprotein was undetectable by serum protein electrophoresis and immunofixation. Urine immunofixation was negative for monoclonal protein. Serum‐free kappa chain level was normal, with a normal kappa/lambda light chain ratio. The blood film showed circulating plasma cells with plasmablast morphology (Figure 1A). By flow cytometry, these cells were negative for CD45, CD5, CD10, CD19, CD20, CD56, CD33, CD23, and immunoglobulin (Figure 1C). The bone marrow aspirate showed background staining and 90% plasma cells with atypical binucleated forms (Figure 1B). CD138‐cell FISH showed a gain at 1q21 (69%), deletion of the *TP53* gene at 17p13 (10%), and no evidence of immunoglobulin rearrangement. Trephine biopsy showed 80%–90% plasma cells, which were positive for CD138, cyclin D1, MUM1, and negative for kappa and lambda staining (Figure 2). The PET/CT showed multifocal avid bone disease. He was diagnosed with non‐secretory multiple myeloma (NSMM) (stage ISS 3, R‐ISS 3), non‐producing phenotype, and primary plasma cell leukaemia. He had a poor response to multiple lines of treatment (bortezomib/ cyclophosphamide/ lenalidomide/ dexamethasone (VCRD), VRD‐PACE (cisplatin/ doxorubicin/ cyclophosphamide/ etoposide), melphalan autograft transplantation, carfilzomib/ lenalidomide/ dexamethasone (KRD), isatuximab/ pomalidomide/ dexamethasone and teclistamab) and sadly passed away within 3 years of diagnosis.

**FIGURE 1:**
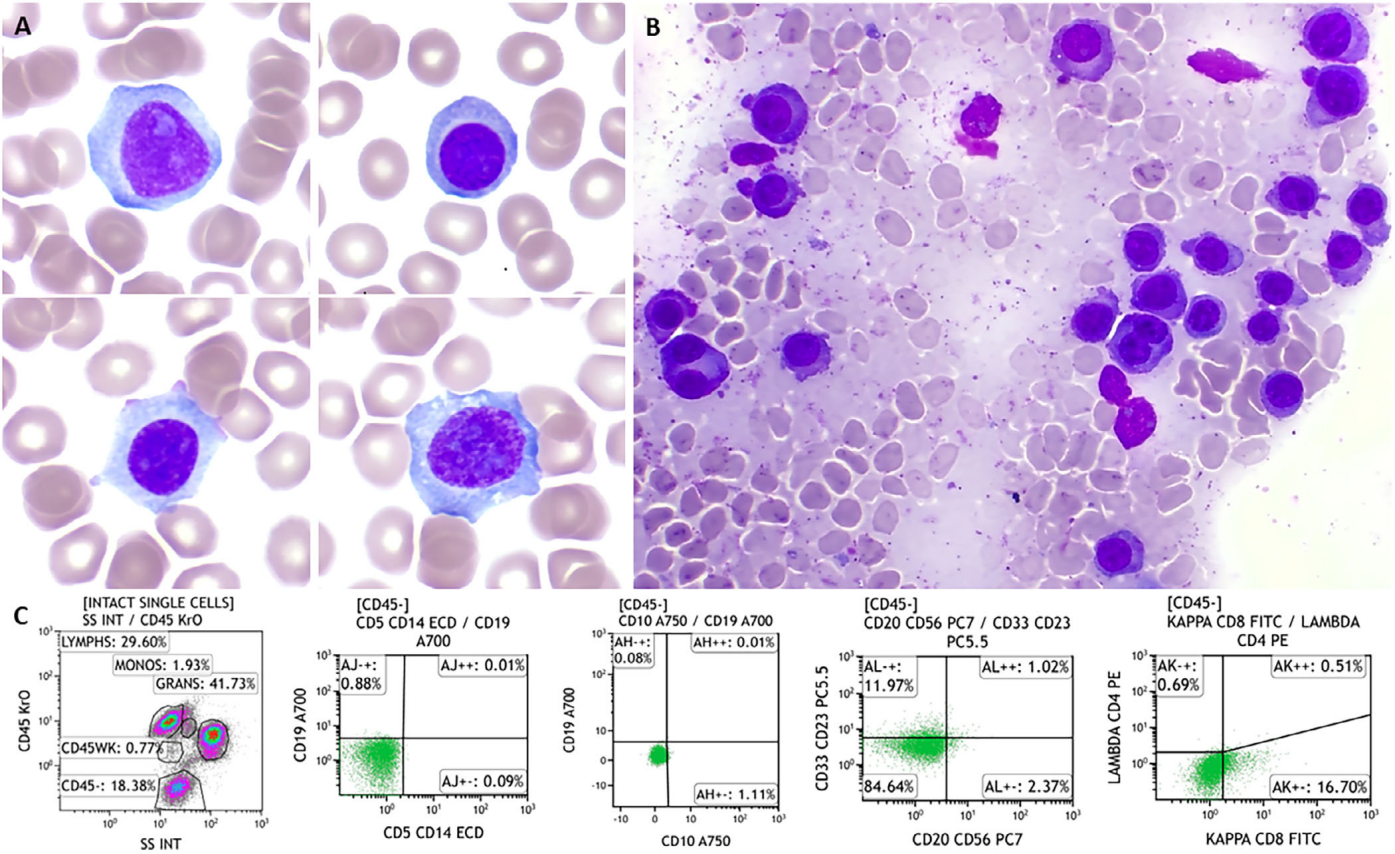
(A) Blood film (×100 objective) showed large atypical plasma cells, some with prominent nucleoli and plasmablast morphology; (B) bone marrow aspirate (×40 objective) showed background staining and heavy infiltration of plasma cells with atypical binucleated forms; (C) peripheral blood immunophenotyping showing a population (green colour) of cells that are CD45‐CD5‐CD19‐CD20‐CD56‐CD33‐CD23‐Ig‐.

**FIGURE 2:**
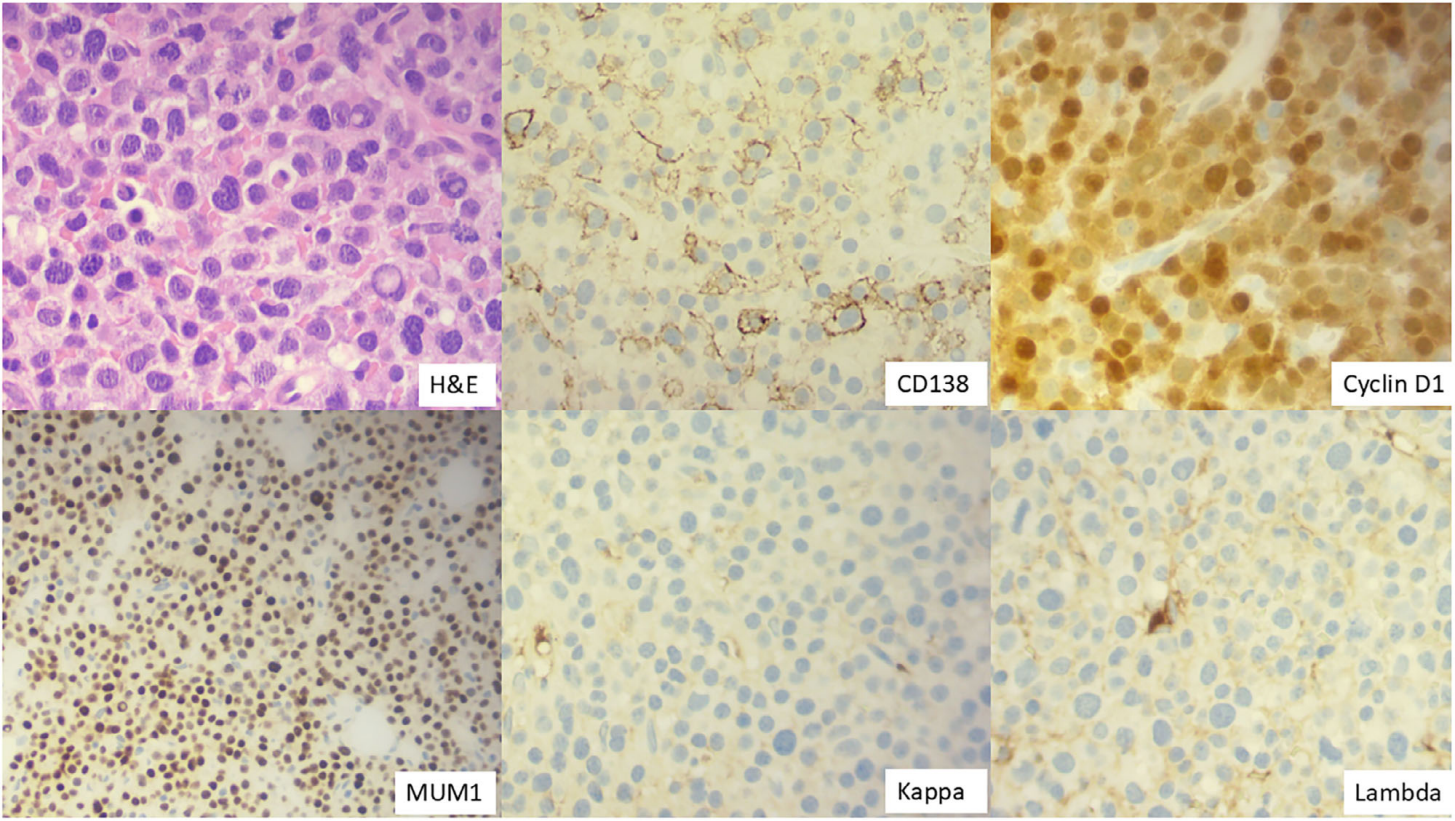
Bone marrow biopsy H&E stain (×40 objective). Immunostaining was positive for CD138 (×40 objective), cyclin D1 (×40 objective), MUM1 (×20 objective), and negative for kappa and lambda staining (×40 objective).

NSMM is a rare subtype of myeloma accounting for 1%–3% of all myeloma cases, in which there is no detectable monoclonal protein secretion by neoplastic plasma cells [1]. Non‐secretors can produce but have defects in secreting immunoglobulins. Non‐producers are unable to synthesize immunoglobulins, and no light‐chain‐restricted plasma cells are detectable by immunohistochemistry, flow cytometry, or in situ hybridization. It is a challenge to detect disease early at the asymptomatic (smouldering myeloma) or MGUS stage. Whole‐body functional imaging (PET/CT, MRI) is essential in identifying myeloma‐defining lesions, risk stratification and assessing treatment response by monitoring the reduction in metabolic activity or changes in diffusion properties. B‐cell maturation antigen (BCMA) is highly expressed on plasma cells. Soluble BCMA, a blood marker, correlates strongly with the total amount of bone marrow plasma cells [2]. The novel soluble BCMA assay could be an essential tool for monitoring treatment response [2], therefore, reducing the need for multiple bone marrow evaluations. Recognizing NSMM, a rare subtype of myeloma, is important to avoid misdiagnosis. Here, we report a rare case of NSMM with a non‐producing phenotype, presented as plasma cell leukaemia.

## Author Contributions

K.X. wrote up the manuscript. K.X. and I.P. critically revised the final version of the manuscript.

## Funding

The author have nothing to report.

## Ethics Statement

The authors have nothing to report.

## Conflicts of Interest

The authors declare no conflicts of interest.

## Data Availability

The data that support the findings of this study are available from the corresponding author upon reasonable request.
